# Biosorption Ability of Pharmaceutically Active Compounds by *Anabaena* sp. and *Chroococcidiopsis thermalis*

**DOI:** 10.3390/molecules29184488

**Published:** 2024-09-21

**Authors:** Jerzy Pogrzeba, Anna Poliwoda

**Affiliations:** Department of Analytical Chemistry, Faculty of Chemistry and Pharmacy, University of Opole, Pl. Kopernika 11a, 45-040 Opole, Poland

**Keywords:** pharmaceuticals, biosorption, cyanobacteria, *Anabaena* sp., *Chroococcidiopsis thermalis*

## Abstract

Drug overuse harms the biosphere, leading to disturbances in ecosystems’ functioning. Consequently, more and more actions are being taken to minimise the harmful impact of xenopharmaceuticals on the environment. One of the innovative solutions is using biosorbents—natural materials such as cells or biopolymers—to remove environmental pollutants; however, this focuses mainly on the removal of metal ions and colourants. Therefore, this study investigated the biosorption ability of selected pharmaceuticals—paracetamol, diclofenac, and ibuprofen—by the biomass of the cyanobacteria *Anabaena* sp. and *Chroococcidiopsis thermalis*, using the LC-MS/MS technique. The viability of the cyanobacteria was assessed by determining photosynthetic pigments in cells using a UV–VIS spectrophotometer. The results indicate that both tested species can be effective biosorbents for paracetamol and diclofenac. At the same time, the tested compounds did not have a toxic effect on the tested cyanobacterial species and, in some cases, stimulated their cell growth. Furthermore, the *Anabaena* sp. can effectively biotransform DCF into its dimer.

## 1. Introduction

The global demand for medicines is systematically growing and applies primarily to highly economically developed countries, including Poland. Economic growth and population ageing increase the demand for healthcare and pharmaceutically active compounds (PhACs). Many people use over-the-counter (OTC) drugs on their own, without a formal prescription, because they are widely available. In Poland, about 54% of the pharmacy market consists of OTC (over-the-counter) drugs, of which 57% are painkillers and non-steroidal anti-inflammatory drugs [1]. Paracetamol, ibuprofen, and aspirin are widely used to manage pain, including fever, headache, musculoskeletal pain, and menstrual cramps. This situation significantly threatens our ecosystem because improperly protected and managed PhACs enter the environment from pharmaceutical industries, hospitals, and domestic and veterinary effluents.

Consequently, pharmaceuticals are the most common micropollutants detected in water resources, usually harming aquatic organisms and negatively affecting environmental water quality [2,3,4,5,6,7]. Furthermore, conventional wastewater treatment plants (WWTPs), which involve biotic and abiotic processes, do not always effectively remove all pharmaceutical xenobiotics from wastewater [8,9]. Therefore, the environmental level of drug contamination varies widely and depends heavily on the degree of industrialisation. Moreover, according to the monitoring data obtained from surface water samples over the last ten years in Europe, the highest detected concentrations for diclofenac, ibuprofen, and paracetamol reached 50 µg L^−1^, 300 µg L^−1^, and 230 µg L^−1^, respectively [10,11,12,13]. Therefore, there is an urgent need to understand PhACs’ environmental occurrence, fate, and degradation pathways in order to provide a comprehensive risk assessment of drug residues in aqueous environments. Furthermore, green bioremediation processes are an attractive alternative for transforming PhACs into low-toxicity products and achieving further mineralisation.

In this case, biosorbents (microorganisms) such as fungi, algae, yeasts, and bacteria can be suitable for efficient environmental cleaning technologies with higher productivity and lower costs. Physical, chemical, and ionic interactions are involved in the sorption/adsorption of different contaminants by living cells of microorganisms [8,14,15,16,17,18,19]. Also, cyanobacteria can bioadsorb and effectively biodegrade environmental pollutants such as phosphonates [20]. Compared to bacteria and fungi, cyanobacteria perform better in remediation, since their cell walls contain polysaccharides, which can adsorb various micropollutants, especially organic compounds. Characterisation of metal–cyanobacteria sorption reactions has demonstrated that cyanobacterial surfaces are complex structures with distinct surface layers, each with unique molecular functional groups and metal-binding properties [19,21,22,23,24,25].

Furthermore, as a whole cell, cyanobacteria may also be active biocatalysts during the process of pharmaceutical biodegradation. For example, the cyanobacterium *Phormidium* sp. helped in the biosorption of phenol [26], whereas *Fischerella* sp. enabled the removal of methyl parathion with around 80% efficiency [27]. Also, the dead biomass of cyanobacteria has equal sorption potential compared to living cells and has already been effectively applied to treat industrial wastewater [28].

Although information regarding the correlation between the presence of cyanobacteria in the ecosystem and the concentration of PhACs is very preliminary, this group of chemical substances is visible in environments inhabited by those microorganisms. Research on the efficiency of cyanobacterial bioremediation in reducing nutrient levels in eutrophic lakes with respect to the presence and possible use of PhACs as a source of nutrient ingredients is limited; therefore, structural questions arise: What are the potential roles of PhACs in cyanobacterial metabolism? Can cyanobacteria take part in PhACs’ biodegradation processes in the environment? What are the metabolites produced during this process? Only a few studies have explored the effects of painkillers and non-steroidal anti-inflammatory drugs on cyanobacteria [29,30,31,32]. For example, exposure to paracetamol (in the concentration range of 25–150 mg L^−1^) showed toxic responses on the test organism (the cyanobacteria *Nostoc muscorum*) by generating oxidative stress [29].

Meanwhile, at 1–1000 µg L^−1^ concentrations, ibuprofen stimulated the growth of the cyanobacterium *Synechocystis* sp. over five days of exposure [32]. Therefore, this investigation is unique because the influence of anti-inflammatory drugs on cyanobacteria’s growth and biosorption ability has not yet been investigated widely. Furthermore, exploring alternative, environmentally sustainable methods for removing pharmaceuticals from water is of urgent necessity. With their unique cellular structure and biochemical properties, cyanobacteria offer a promising solution. Unlike traditional biosorbents that focus primarily on heavy metals, using cyanobacteria for the removal of PhACs represents a novel approach. The ability of cyanobacteria to both adsorb and potentially biotransform such compounds has not been widely investigated, especially concerning these common pharmaceuticals. Therefore this study aims to evaluate the biosorption and biotransformation abilities of two cyanobacterial species, Anabaena sp. and *Chroococcidiopsis thermalis*, in removing paracetamol, diclofenac, and ibuprofen from aqueous solutions. By understanding these interactions, we aim to contribute to developing more effective bioremediation strategies for managing pharmaceutical pollution in water systems. Furthermore, changes in cyanobacterial populations due to drug residues can have broader ecological consequences. Cyanobacteria are primary producers and play a key role in aquatic food webs. Alterations in their abundance and distribution can impact higher trophic levels and overall ecosystem dynamics [33]. In particular, the potential for pharmaceuticals to contribute to cyanobacterial blooms is a critical concern, as these blooms can lead to harmful algal events that disrupt aquatic ecosystems, deplete oxygen levels, and produce toxins. Therefore, understanding how pharmaceuticals such as paracetamol, diclofenac, and ibuprofen affect cyanobacterial growth is essential for assessing the risks to aquatic systems and developing effective bioremediation strategies to mitigate these impacts.

Cyanobacteria are photosynthetic microorganisms that rely heavily on chlorophyll for photosynthesis. The level of chlorophyll within cyanobacterial cells can provide significant insights into their physiological status and environmental responses. For instance, reduced chlorophyll levels may indicate stress responses to adverse conditions such as high temperatures, nutrient deficiencies, or exposure to chemical stressors. Conversely, elevated chlorophyll levels and protective mechanisms like increased carotenoid production suggest that cyanobacteria adapt effectively to environmental stressors. [34]. Moreover, monitoring chlorophyll content is a helpful tool for assessing cyanobacterial populations’ vitality and stress levels. However, excessive cyanobacterial growth, often indicated by high chlorophyll levels, can lead to adverse environmental outcomes, such as algal blooms, oxygen depletion, and the production of harmful toxins that affect both aquatic life and human health [35,36].

It is worth noting that most of the studies published thus far typically focus on experiments conducted using only one species of microorganism. While these studies provide valuable insights, they may not capture the diversity of responses that different species can exhibit. By examining two distinct cyanobacterial species, *Anabaena* sp. and *Chroococcidiopsis thermalis*, this study offers a comparative perspective on the biosorption and biotransformation of pharmaceuticals. This dual-species approach not only enhances our understanding of cyanobacteria in bioremediation but also highlights the importance of investigating multiple species to identify the most effective biosorbents for environmental applications.

## 2. Results and Discussion

### 2.1. Effects of Paracetamol, Diclofenac, and Ibuprofen on the Growth of Cyanobacteria

**Figure 1:**
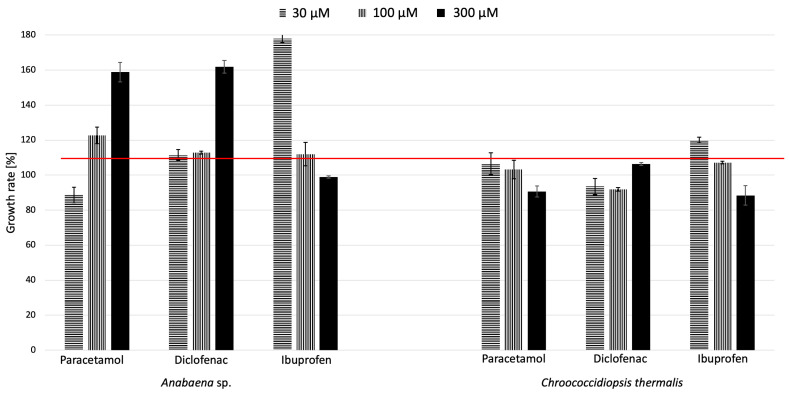
The effects of paracetamol, diclofenac, and ibuprofen on the growth rate factor of the studied cyanobacteria species (21 days of cultivation; red line—growth factor for control, *n* = 3).

To study the effects of specific pharmaceuticals, two freshwater cyanobacteria species, *Anabaena* sp. and *Chroococcidiopsis thermalis*, were cultivated for 3 weeks in the presence of paracetamol, diclofenac, and ibuprofen at three different concentration levels (30 µM, 100 µM, and 300 µM). The three concentrations of pharmaceuticals tested in this study were chosen to cover a range of both environmentally relevant and experimental levels. The 30 µM concentration approximates the higher end of pharmaceutical concentrations reported in surface waters, particularly in areas with significant contamination. The intermediate concentration of 100 µM was included to observe cyanobacterial responses under moderate exposure conditions, while the 300 µM concentration, though exceeding typical environmental levels, allowed for the investigation of the cyanobacteria’s capacity to handle extreme contamination scenarios. This range of concentrations provides a comprehensive understanding of how cyanobacteria perform under varying degrees of pharmaceutical pollution, from more common levels to extreme cases. Then, the growth rate factor (GRF) was determined based on chlorophyll content (Figure 1), where the red line indicates the level of 100%, representing cell growth in the control sample—without pharmaceuticals. The bars above this line indicate enhanced growth, while those below indicate inhibited growth.

At first glance, the difference in the sensitivity of the tested cyanobacteria species to the presence of the examined pharmaceuticals in their environment is evident. For *Anabaena* sp., PAR and DCF generally enhanced growth at all tested concentrations, with DCF exhibiting the highest growth factor at 300 µM (162%). Contrastingly, IBF had a growth-enhancing effect at lower concentrations but inhibited growth at the highest concentration tested. In the case of *Chroococcidiopsis thermalis*, PAR consistently showed a growth-enhancing effect. However, DCF tended to inhibit growth, as indicated by a growth factor consistently below the baseline. As with *Anabaena* sp., IBF enhanced growth at lower concentrations but exhibited inhibitory effects at higher concentrations. PAR was generally beneficial for growth in both cyanobacteria types. DCF, on the other hand, showed a differential impact, stimulating growth in *Anabaena* sp. but inhibiting it in *Chroococcidiopsis thermalis*. IBF demonstrated a concentration-dependent effect in both cyanobacteria, promoting growth at lower concentrations and leading to inhibition at higher concentrations.

Furthermore, *Chroococcidiopsis thermalis* displayed conservative responses, with only a minor reduction in growth. The effects of these compounds seemed to be concentration-dependent, suggesting a threshold at which the promotion of growth shifts to inhibition. This pattern indicates that higher concentrations of compounds, such as ibuprofen, might exert cytotoxic effects, diminishing the growth of cyanobacteria. In conclusion, the results indicate that the impact of different pharmaceuticals on the growth of cyanobacteria can vary significantly depending on the cyanobacterial species, the specific pharmaceutical, and its concentration.

**Figure 2:**
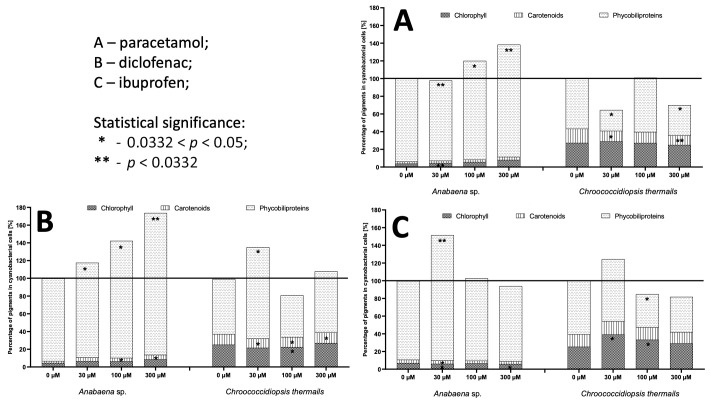
The impact of (**A**) paracetamol, (**B**) diclofenac, and (**C**) ibuprofen on the distribution of photosynthetic pigments in cyanobacterial cells after 21 days of cultivation. The red line represents the total pigment content in control samples. The asterisks on the bars indicate statistically significant changes in pigment content compared to the corresponding control group. The number of asterisks represents the level of statistical significance.

Also, the levels of phycobiliproteins and carotenoids in cyanobacterial cells can provide critical information about cyanobacteria’s health, functionality, and adaptation to environmental conditions [37,38,39]. As a result, the next step examined the impact of the pharmaceuticals in the culture medium on the contents of photosynthetic pigments—chlorophylls, carotenoids, and phycobiliproteins—in the tested cyanobacteria (Figure 2).

Phycobiliproteins are auxiliary pigments involved in the photosynthesis of cyanobacteria. Their levels can inform us about the ability of these organisms to utilise sunlight for photosynthesis. A high level of phycobiliproteins may indicate a good capacity for capturing light energy [37,40]. Meanwhile, elevated levels of carotenoids, such as carotenes, may respond to environmental stress, including excess light or exposure to harmful factors like UV radiation. Carotenoids act as antioxidants and help protect cells from oxidative damage [38,39].

Considering our studies, the phycobiliprotein levels of *Anabaena* sp. significantly corresponded with the tested pharmaceuticals’ effects on chlorophyll content and growth factors. At 100 µM diclofenac or paracetamol, he photosynthetic pigments of this cyanobacterium were significantly increased (at *p* < 0.05) compared to the control. Also, the total carotenoid content was higher when exposed to diclofenac. However, in the case of ibuprofen, an increase in carotenoid biosynthesis was only observed at the lowest concentrations. Furthermore, increasing the IBF concentration in the culture medium resulted in a decrease in carotenoids in cells of this blue–green alga. In the case of the species *Chroococcidiopsis thermalis*, more significant changes in the ratio of photosynthetic pigments in cells were noticeable, indicating a reduced amount of phycobiliproteins. There was a more frequent, statistically significant increase in the concentration of chlorophylls and carotenoids (diclofenac 30–300 µM and ibuprofen 30 µM and 100 µM, respectively). This phenomenon may indicate a different reaction mechanism of the studied species to stress factors. In a few cases, the total amount of pigments in cells exceeded the concentration in the control sample, proving their increased production in the presence of stress factors in the medium. Therefore, it can be concluded that photosynthetic pigments play a role in directly or indirectly counteracting the stress factor. Similar relationships have already been shown in the literature regarding the effects of phosphonates and boronic acids on cyanobacterial cells [41,42].

Only two studies in the literature explore the effects of selected pharmaceuticals on photosynthetic prokaryotes, which play a crucial role in food chains. One study examined the impact of paracetamol on the growth of the cyanobacterium *Nostoc muscorum* [30], while the other investigated ibuprofen’s influence on *Synechocystis* sp. PCC6803 [43]. In the former paper, exposure to paracetamol (at concentrations of 25 mg L^−1^, 50 mg L^−1^, 75 mg L^−1^, 100 mg L^−1^, 125 mg L^−1^, and 150 mg L^−1^) showed toxic responses in *Nostoc muscorum*, inducing oxidative stress. In contrast, ibuprofen strongly stimulated the growth of *Synechocystis* sp. PCC6803 at all concentrations, with a 72% increase at 10.0 μg L^−1^. In the latter study, the highest tested concentration of the pharmaceutical pollutant was 1000 µg L^−1^. Both studies used incubation times of 5 or 9 days to investigate the effects of the selected pharmaceuticals. For comparison, in this study, results were derived from 21 days of cultivation.

Furthermore, *Anabaena* sp., *Chroococcidiopsis thermalis*, *Synechocystis* sp., and *Nostoc muscorum* are all types of cyanobacteria, but they differ in terms of their characteristics, morphology, ecological roles, and habitat preferences. *Anabaena* sp. is a filamentous cyanobacterium that forms long chains of cylindrical cells. It possesses specialised cells called heterocysts for nitrogen fixation. *Chroococcidiopsis thermalis* is a unicellular cyanobacterium that is often found in extreme environments. It typically exists in small colonies, rather than as single cells. *Synechocystis* sp. consists of unicellular or tiny colonial cyanobacteria that do not form long chains and lack specialised nitrogen-fixing cells like heterocysts. *Nostoc muscorum* is a filamentous cyanobacterium that forms gelatinous colonies or mats. It has heterocysts for nitrogen fixation and can establish symbiotic relationships with plants.

Moreover, *Anabaena* sp. is commonly found in freshwater environments, especially in nutrient-rich bodies of water, where visible blooms can form. *Synechocystis* sp. is widespread in various freshwater habitats, including biofilms and terrestrial environments. *Chroococcidiopsis thermalis* is often located in extreme environments such as hot springs, deserts, and rocky substrates, where it can withstand high temperatures and desiccation. *Nostoc muscorum* is generally encountered in terrestrial settings like soil, rocks, and mosses, but it can also be found in aquatic environments. The prevalence of each of these cyanobacteria depends on the habitat’s suitability for their growth and survival.

In summary, all of the studied xenopharmaceuticals significantly impacted the metabolism of the tested cyanobacteria species, with effects varying depending on the concentration. The most notable metabolic changes were observed in *Chroococcidiopsis thermalis*, where these changes were distinct from the relatively stable growth coefficient. These findings also highlight the importance of stress response mechanisms, as both phycobiliproteins and carotenoids were synthesised more intensively by cells from both species in experimental cultures. The different effects of drug residues in the environment on *Anabaena* sp. and *Chroococcidiopsis thermalis* can be attributed to several factors. Different cyanobacterial species may exhibit varying sensitivity to specific drugs or drug residues. Their metabolic and physiological characteristics can differ, leading to diverse responses to the same substances. *Anabaena* sp. and *Chroococcidiopsis thermalis* might possess distinct metabolic pathways and enzyme systems.

Consequently, drug residues can disrupt specific metabolic processes, resulting in different responses based on the underlying metabolic mechanism. Furthermore, drug residues can exert their effects through various mechanisms, such as disrupting cellular processes, inhibiting enzyme activity, or compromising cell membrane integrity. *Anabaena* sp. and *Chroococcidiopsis thermalis* appear to have distinct stress response mechanisms. Drug residues can induce stress in cyanobacteria, with the specific stress response pathways activated varying between species. Additionally, the environmental conditions in which these cyanobacteria grow can affect their responses to drug residues. The specific reasons for the divergent effects of drug residues on *Anabaena* sp. and *Chroococcidiopsis thermalis* likely involve a combination of these factors. Understanding the precise mechanisms behind these different responses requires further research and investigation into the specific drugs and environmental conditions involved.

### 2.2. Biosorption Ability

**Figure 3:**
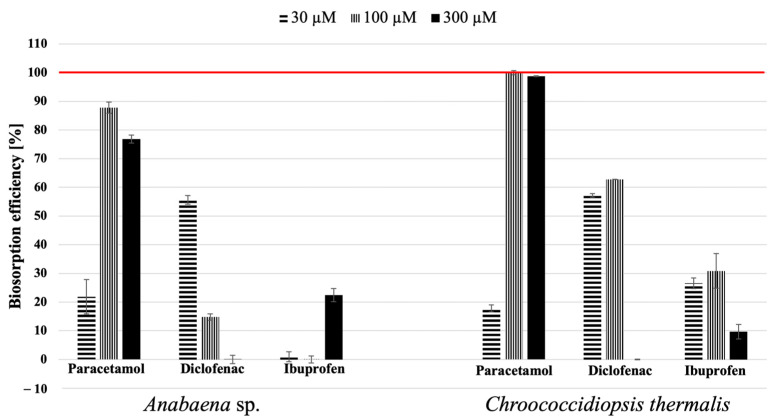
The biosorption efficiency of individual xenopharmaceuticals from the growth medium on the 21st day of cultivation. A red line indicates complete drug removal (100%). The error bars represent the standard error from at least nine replicates.

The main objective of this study was to examine the effectiveness of *Anabaena* sp. and *Chroococcidiopsis thermalis* cells for removing paracetamol, diclofenac, and ibuprofen from aqueous culture media. The samples extracted from the post-culture media and cell surfaces after 21 days of cultivation were analysed using the LC-MS/MS technique to investigate the biosorption process. Reference samples were prepared using BG11 medium with specified concentrations of the pharmaceuticals. The influence of the initial pharmaceutical concentration on the biosorption process of the tested cyanobacteria is shown in Figure 3. Paracetamol had the highest removal factor among the tested pharmaceuticals, considering both species of cyanobacteria. The decrease in sorption efficiency in the case of paracetamol and diclofenac was associated with an increase in phycobiliprotein contents in the thylakoid membranes of cyanobacterial *Anabaena* sp. cells. A particular case is represented by the effect of paracetamol at a concentration of 30 µM, where the removal efficiency by both species was about 20%. This efficiency significantly increased at the other two tested concentrations (100 µM and 300 µM), with removal efficiencies reaching 90% for *Anabaena* sp. and 100% for *Chroococcidiopsis thermalis*.

Interestingly, when exposing the tested cyanobacteria to paracetamol concentrations at a level similar to that in the environment, the biosorption ability of the studied blue–green algae was approximately 100%. It is possible that, in the case of cyanobacteria and the influence of drugs on their growth, there is a concentration of pharmaceuticals that generally does not exhibit a toxic effect, at which these microorganisms can effectively cooperate. This applies, among other things, to the environmental concentrations at which the tested drugs were determined. However, over time, an increase in drug concentration may lead to the inhibition of growth or alterations in the metabolism of cyanobacteria. This can affect metabolic processes such as photosynthesis and respiration, leading to changes in metabolic efficiency (like the biological effect observed at a concentration of 30 µM) until the cyanobacteria develop resistance to these substances by activating specific defence mechanisms within the cells to counteract the negative impact of the pharmaceuticals (observed effect at concentrations of 100 µM and 300 µM).

Furthermore, *Ch. thermalis* deserves special attention for its moderate biosorption capability while having a comparatively minor impact on cell growth and metabolism compared to *Anabaena* sp., the other tested species. *Anabaena* sp. and *Chroococcidiopsis thermalis* may have distinct metabolic pathways and enzyme systems, potentially explaining their different biosorption behaviours [44,45,46,47,48]. Drug residues can interfere with specific metabolic processes, leading to varied responses. *Anabaena* sp. is a filamentous cyanobacterium, forming long chains or filaments of cells, and is commonly found in freshwater environments such as lakes, ponds, and slow-moving rivers.

In contrast, *Chroococcidiopsis thermalis* is unicellular or forms small colonies of cells and can be found in extreme environments like hot springs, deserts, and rocky substrates. This adaptability to harsh conditions, including high temperatures and desiccation (drying out), sets *Chroococcidiopsis thermalis* apart, while *Anabaena* sp. typically exists in more temperate aquatic habits. These differences reflect cyanobacteria’s diverse nature and ability to adapt to various ecological niches.

Moreover, an interesting result was observed in the biosorption of ibuprofen. Both studied cyanobacteria species removed it less effectively, not exceeding 30%. Additionally, it can be observed that *Anabaena* sp. removed IBF more efficiently at concentrations of 30 µM and 300 µM, whereas *Chroococcidiopsis thermalis* was more effective at the low and intermediate concentrations (30 µM and 100 µM). Therefore, the relationship regarding the effectiveness of removing this xenopharmaceutical cannot be indicated. Generally, the disrupting factor (tested pharmaceuticals) determines different cyanobacteria’s biological responses. The difference in the toxicity of paracetamol, diclofenac, and ibuprofen to the natural environment depends on several factors, including their chemical properties, environmental persistence, and the sensitivity of the organisms inhabiting it. Paracetamol is a relatively low-risk pharmaceutical in terms of its environmental impact. It is known to break down relatively quickly in the environment, and its toxicity to aquatic organisms is generally considered to be low. However, its presence in surface waters can still have localised effects on aquatic life. Diclofenac has gained notoriety for its harmful environmental effects. In aquatic environments, diclofenac adversely affects aquatic organisms, whereas ibuprofen’s presence can harm fish and other aquatic life [34]. It is important to note that the toxicity of these pharmaceuticals can vary depending on factors such as concentration, exposure duration, and the specific species of organisms present in the environment. Efforts are being made to reduce the environmental impact of pharmaceuticals, including better wastewater treatment and responsible medication disposal practices.

### 2.3. Interaction of Tested Pharmaceuticals with the Cyanobacterial Cell Surface

**Figure 4:**
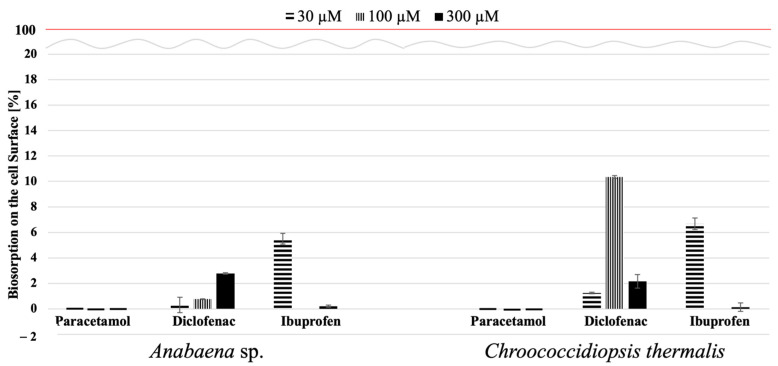
The amount of xenopharmaceutical bioadsorbed on the cell surface on the 21st day of cultivation, expressed as a percentage. A red line indicates complete drug removal (100%). The error bars represent the standard error from at least nine replicates.

The proposed advantage of using active biosorbents in the form of cyanobacteria lies in their ability to reduce the concentration of tested compounds in their environment and their potential for metabolising them. Therefore, in the next step, we investigated whether these compounds could be deposited on the cell surface after demonstrating that the two freshwater-tested cyanobacteria species could remove PAR and DCF from the culture medium. Figure 4 presents the results as a percentage of identified drugs on the cell’s surface with respect to the initial concentration of substances in the medium.

It can be seen that the adsorption of the tested xenopharmaceutical substances, primarily ibuprofen and diclofenac, on the cell surface of the cyanobacteria was relatively low, not exceeding 10% compared to the control sample Furthermore, no paracetamol was detected on the surface of the tested cyanobacteria species’ cells. Therefore, it seems that the adsorption process is not the primary mechanism for removing these pharmaceuticals by cyanobacteria.

At this point, the question arose whether cyanobacteria could utilise pharmaceuticals, such as those tested in this study, as a nutrient source and subsequently incorporate them into their metabolism. Therefore, additional experiments were performed, where the pharmaceuticals were the only carbon source available in the medium. The obtained results demonstrate that paracetamol and diclofenac caused a slight inhibition of cyanobacterial growth (approximately 80% of the control) but did not lead to cell death, and the cyanobacteria (both *Chroococcidiopsis thermalis* and *Anabaena* sp.) were able to metabolise these compounds. In contrast, ibuprofen led to complete cell death, indicating a different interaction mechanism. These findings confirm that certain cyanobacteria can metabolise certain pharmaceuticals, supporting the hypothesis that cyanobacteria can use them as a carbon source, integrating them into their metabolic pathways.

Furthermore, these results indicate that live cyanobacteria not only adsorb pharmaceuticals but also metabolise them into less toxic forms. This is a significant advantage over passive biosorbents, which can only adsorb contaminants without breaking them down. When passive biosorbents degrade or die, they risk releasing the adsorbed substances into the environment. In contrast, the cyanobacteria used in this study metabolised the compounds, reducing the likelihood of re-releasing harmful contaminants after cell death. This finding is consistent with previous research demonstrating that cyanobacteria can degrade organic compounds, including the ability to obtain essential elements such as phosphorus from xenobiotics by breaking down phosphonates or metallic forms—e.g., nanoparticles from metal ions [49,50,51,52]. It can be concluded that the decreased concentrations of the tested pharmaceuticals from the post-culture medium resulted from cyanobacterial biotransformation and not just deposition on the cell surface, as in the case of metals. Therefore, the use of live cyanobacteria as active biosorbents offers a significant environmental advantage, as they do not merely bind contaminants but also transform them into less harmful products, thereby minimising the risk of environmental contamination following cell death.

### 2.4. Identification of Analgesics and Their Metabolites in a Post-Culture Medium Using the MS/MS Technique

**Figure 5:**
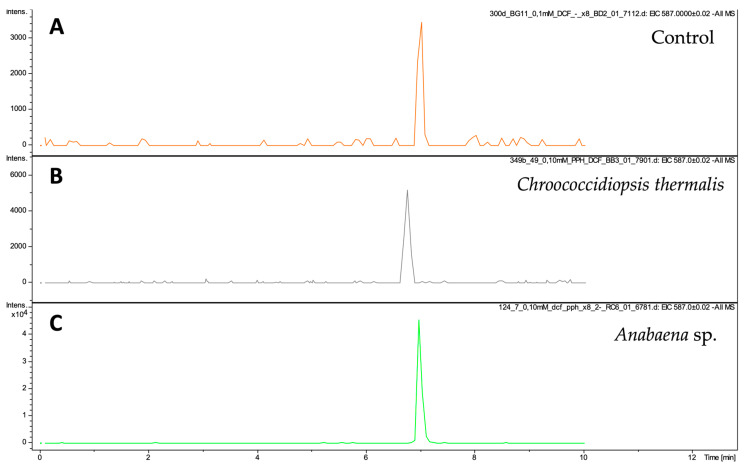
The EIC of *m*/*z* ion 587.02 detected in samples of the control sample (BG11 medium with 100 µM diclofenac and day 21 post-culture media of *Ch. thermalis* and *Anabaena* sp. subjected to the influence of 100 µM DCF (*n* = 3, RSD < 10%).

In the tested post-culture medium, not only was the disappearance of the signal from the tested pharmaceuticals reviewed, the possibility of metabolites of the tested substances being present in the samples was also verified. However, this task turned out to be very complex and complicated. In the experimental data, it was noted that the post-culture medium of *Anabaena* sp. subjected to the action of diclofenac showed the significant presence of a compound with an *m*/*z* value of 587.01 (Figure 5). In the control sample and post-culture media containing DCF and incubated with *Ch. thermalis*, the signal intensity of *m*/*z* ion 587.01 was very low. However, a markedly higher response for *m*/*z* ion 587.01 was observed in the culture fluids of Anabaena sp., indicating the presence of a DFC metabolite.

**Figure 6:**
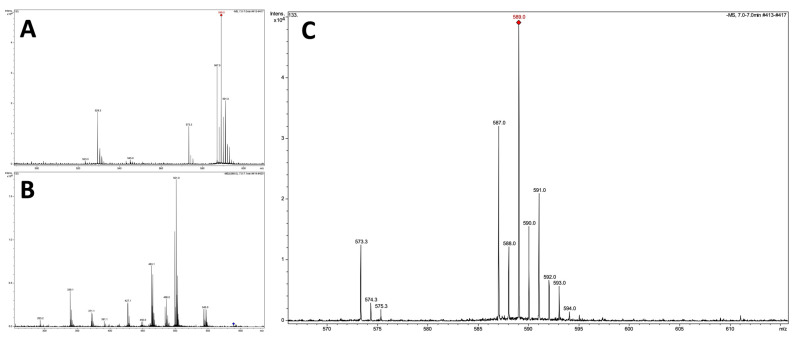
MS and MS/MS spectra of the dissected compound with the *m*/*z* ion value 587.01, determined in the post-culture medium of *Anabaena* sp. containing diclofenac as a studied xenopharmaceutical. (**A**) The MS spectrum of the dissected compound. (**B**) The MS/MS spectrum of the dissected compound. (**C**) An enlargement of (**A**).

This hypothesis proved to be correct, as confirmed by the results from the MS and MS/MS spectral analyses, which are shown in Figure 6. It turned out that the compound of interest is a known DCF metabolite—its dimer, a diclofenac-2,5-iminoquinone (DCF-2,5-IQ). For example, such a compound was also formed during the electro-oxidation of DCF on a boron-doped diamond electrode, and especially in the oxidation reaction of diclofenac in the presence of iron(II) octacarboxyphthalocyanine and myeloperoxidase [53,54,55]. This result seems to be very interesting because, most likely, the presence of the naturally occurring porphyrins and enzymes containing metal ions in cyanobacterial cells enable cyanobacteria to participate in the redox reactions and transform 5-hydroxy-diclofenac (DCF) into its metabolite, the aforementioned dimer. Furthermore, it should be noted that this phenomenon, when cyanobacteria can biotransform diclofenac into a dimer form, has never been described in the literature before. Until now, the transformation of 5-hydroxy-diclofenac into DCF-2,5-IQ has been proven only as a result of experiments performed under laboratory conditions (in vitro studies) with selected catalysts (e.g., iron(II) octacarboxyphthalocyanine) and oxidants (e.g., hydrogen peroxide (H_2_O_2_) and NaIO_4_). What is also interesting is the fact that only Anabaena exhibited this property among the tested microorganisms. In the case of *Ch. thermalis*, the presence of the dimer was not observed at all. These differences may also directly result from the proven greater sensitivity of the cyanobacterium *Anabaena* sp. to the presence of diclofenac in the medium compared to *Ch. thermalis*.

Although research on the toxicity of diclofenac dimers to various organisms is limited, existing studies suggest that these dimers may be less toxic than diclofenac itself. In one study, the toxicity of a diclofenac dimer was tested on *E. coli* bacteria, showing a significant negative effect only at concentrations above 6 μM [54]. Interestingly, the same study demonstrated that the dimer was less toxic to cancer cells when compared to diclofenac. Another study indicated that diclofenac dimers, due to their large molecular size—approximately 600 daltons—are less likely to penetrate biological membranes and interact with enzymes and intracellular structures [56]. This suggests a reduced potential for toxic effects on organisms. Moreover, cyanobacteria can metabolise diclofenac, likely as a protective mechanism against its harmful effects, converting it into less toxic forms. This metabolic transformation by cyanobacteria may reduce the overall toxicity of diclofenac in aquatic environments, although further studies are needed to confirm the environmental safety of the resulting dimers.

Unfortunately, looking for potential metabolites of paracetamol and ibuprofen did not yield the desired results. The analysis of the culture fluids did not prove the presence of any metabolites known from the literature. Most likely, conducting experiments on a larger scale (e.g., in bioreactors) will provide the opportunity to detect such biotransformation products, which we plan to implement in the future.

## 3. Materials and Methods

### 3.1. Chemicals

The tested pharmaceuticals—paracetamol (PAR), diclofenac (DCF), and ibuprofen (IBF)—and all chemicals used for cyanobacteria cultivation were of analytical grade and were purchased from Merck Sp. z o.o. (Warsaw, Poland). The culture medium with appropriate concentrations of PAR (30 μM, 100 μM, and 300 μM) was prepared by mixing BG11 medium with the right amounts of paracetamol (e.g., 45.5 mg to obtain the concentration of 300 µM) in a 1 L volumetric flask. Due to the hydrophobicity of DCF and IBF, the appropriate amounts of those pharmaceuticals were first dissolved in 1.5 mL of acetone (to increase their solubility) and then dissolved in a 1 L volumetric flask containing BG11 culture medium. To prepare a 300 µM solution, 88.8 mg of diclofenac or 6.19 mg of IBF was weighed. The solvents and chemical reagents used for LC-MS analysis were of LC-MS grade and were obtained from Merck Sp. z o.o. (Warsaw, Poland). Avantor Performance Materials Poland (Gliwice, Poland) supplied the inorganic salts and acids. The water was purified with a Milli-Q-RO4 system (Millipore, Bedford, MA, USA). The BG11 medium was prepared according to the guidelines available for the culture of freshwater, soil, thermal, and marine cyanobacteria [31].

### 3.2. Biosorption Experiments

Batch biosorption experiments were carried out with the use of two freshwater cyanobacteria species: *Anabaena* sp. (CCALA 007) and *Chroococcidiopsis thermalis* (CCALA 049), obtained from the collection of the Botany Institute of the Academy of Sciences of the Czech Republic in Trebon. Cultivations were performed in sterile conical flasks (100 mL) using liquid BG11 medium (ATCC 616). The initial amount of biomass in each culture, expressed as the chlorophyll concentration and determined spectrophotometrically, was 1 mg L^−1^. The growth conditions were 1050 lux light using a 16:8 photoperiod and a temperature of 25 ± 1 °C. For the biosorption tests, the 30, 100, and 300 µM solutions of the studied pharmaceuticals were added to the cultivation medium on the first day of biomass growth. Cultures without the addition of the tested xenobiotics served as control samples (blank samples). Triplicates were used to estimate the levels of all studied pharmaceuticals in the solution. After 21 days of cultivation, the cyanobacteria cells were separated from post-culture media by centrifugation. The obtained cells’ biomass was washed three times with ethyl acetate to isolate any pharmaceuticals that had potentially been adsorbed on the surface of the tested cyanobacteria. The obtained fractions were combined, and then the excess solvent was evaporated in a nitrogen stream. Finally, the solid residue was dissolved in 1 mL of an aqueous methanol mixture (1:1 *v*/*v*) and analysed using the LC-MS/MS technique. In parallel, the post-culture media were stored at –48 °C overnight and lyophilised for 48 h in a freeze dryer (Alpha 1–2 LD plus, Martin Christ, Harz, Germany). In the next stage, ethyl acetate was added to the obtained lyophilised media to isolate the studied pharmaceuticals, and the exact steps were performed with cyanobacteria cells before LC-MS/MS analysis.

The removal efficiency and surface bioaccumulation of the tested xenobiotics by the cyanobacteria were calculated according to Equations (1) and (2):

(1) $${BioE}_{PCM}\left[\%\right]=100\%-\left(\frac{S_{e}}{S_{r}}\cdot 100\right)$$

(2) $${BioE}_{CS}\left[\%\right]=\frac{S_{ec}}{S_{r}}\cdot 100$$

where

BioE_PCM_—efficiency of biosorption from the post-culture medium;
BioE_CS_—efficiency of biosorption on the cell surface;
S_e_—peak area of the studied analytes in extracts of the post-culture medium;
S_ec_—peak area of the studied analytes in the extracts from the cells’ surface;
S_r_—peak area of the studied analytes in the reference sample.

As reference samples, BG11 medium supplemented with the test pharmaceuticals (without cyanobacteria) was incubated under the conditions of the experimental samples. Briefly, the peak areas from the chromatographic analysis of the experimental culture extracts were compared to those of the reference samples and converted to the percentage [%] of substance remaining in the medium.

### 3.3. The Growth and Metabolic Response Studies

The contents of photosynthetic pigments were determined using the method described in the work of Niemczyk et al. [41]. Briefly, the chlorophyll content in the biomass was determined in methanolic extracts. It was been determined after 4, 7, 10, 14, 18, and 21 days of cultivation. In this case, 1 mL of the cell solution was centrifuged (5 min, 13,000 rpm). The sediment of the obtained cells was mixed with MeOH (0.9 mL), shaken, and incubated in the dark (10 min). This procedure was performed twice. Then, the extracts were centrifuged (5 min, 13,000 rpm), and the chlorophyll content was determined at 645 nm and 663 nm using a Rayleigh 2601 UV-VIS spectrophotometer (Beijing, China). The total concentration of chlorophylls was calculated using Equation (3) [57]:

C_Chlorophyll_ [mgL^−1^] = 20.2·A_645_ + 8.02·A_663_ (3)

The growth rate factor (GRF) was determined based on chlorophyll content. The GRF was calculated from the slopes of the growth curves, and the results were converted into percentages and compared to the control (established as 100%).

The total content of carotenoids in cells was determined over 21 days of cultivation experiments. The applied analytical procedure was similar to that for chlorophylls, but dimethylformamide (DMF) was used as an extractant. The carotenoid contents were determined at 461 nm and 664 nm using Equation (4) [58]:

C_Carotenoids_ [mgL^−1^] = (A_461_ − (0.046·A_664_))·4 (4)

Regarding the phycobiliprotein contents, the cell solution (1.5 mL) was collected on the 21st day of the cultivation experiments and centrifuged (10 min, 13,000 rpm) at 4 °C. Then, 0.15 mL of glycerol was added to the obtained cell sediment and mixed for 24 h at −5 °C. Next, 1.35 mL of distilled water was added, shaken, and centrifuged (5 min, 13,000 rpm, temp. 5 °C). The phycobiliprotein contents were determined in the supernatants at 562 nm, 615 nm, and 652 nm. The concentrations of individual phycobiliproteins and total concentrations were calculated using Equations (5)–(8) [59]: 

Phycocyanin (PC):

(5) $$C_{PC}\left[{mgL}^{-1}\right]=\frac{A_{615}-(0.472\cdot A_{652})}{5.34}\cdot 1000$$

Allophycocyanin (APC):

(6) $$C_{APC}\left[{mgL}^{-1}\right]=\frac{A_{652}-(0.208\cdot A_{615})}{5.09}\cdot 1000$$

Phycoerythrin (PE):

(7) $$C_{PE}\left[{mgL}^{-1}\right]=\frac{A_{562}-2.41\cdot \left[C_{PC}\right]-0.849\cdot [C_{APC}]}{9.62}\cdot 1000$$

Total concentration of phycobiliproteins (PBPs):

(8) $$C_{PE}\left[{mgL}^{-1}\right]=C_{PC}+C_{APC}+C_{PE}$$

### 3.4. Statistical Analysis

The cells’ photosynthetic pigment concentrations were calculated using the equations given in Section 3.3 and averaged over three replicates. Statistical significance was analysed in GraphPad Prism 9 using the multiple *t*-test, corrected for multiple comparisons using the Holm–Sidak method. The levels of statistical significance are marked with asterisks, as follows: 0.05 < *p*; * 0.0332 < *p* < 0.05; ** *p* < 0.0332. The remaining calculations were performed using MS Office Excel 2019.

### 3.5. LC-UV-MS/MS Analyses

The obtained extracts were analysed using a Thermo Ultimate 3000 RS HPLC LC-UV-MS/MS (Sunnyvale, CA, USA) system coupled with a micrOTOF-QII mass spectrometer (Bruker Daltonics, Bremen, Germany) with an ESI source. The studied pharmaceuticals were separated on an Accucore C18 column (100 × 2.1 mm) equipped with a C18 pre-column (2 × 2.1 mm), both from Thermo Fisher Scientific (Waltham, MA, USA), using a mobile phase consisting of acetonitrile (A), an aqueous solution containing 0.1% HCOOH and 0.1 mM HCOONH4 (B), and methanol (C). The gradient mode was used with the following parameters: t = 0 min, 10A:80B:10C; t = 1 min, 10A:80B:10C; t = 3 min, 50A: 0B:50C; t = 5 min, 50A: 0B:50C; t = 8 min, 10A:80B:10C; and t = 10 min, 10A:80B:10C. The flow rate was set at 0.3 mL/min. The MS analyses were performed in the positive electrospray ionisation mode (ESI (+)) for paracetamol and negative electrospray ionisation mode (ESI (-)) for diclofenac and ibuprofen. The SRM *m*/*z* characteristic fragmentation pattern for each of the individual PhACs was determined at 152.07/110.1, 294.01/214.0, and 205.13/161.1 for PAR, DCF, and IBF, respectively.

The software platforms Bruker Daltonics Data Analysis 4.0 SP5 and HyStarPP 3.2.44.0 were used for chromatogram conversion and integration, while Excel 2019 was used for regression analysis of the obtained results. GraphPad Prism 9 and Excel 2019 were used to evaluate spectrophotometry data.

## 4. Conclusions

This study demonstrates the potential of cyanobacteria, specifically *Anabaena* sp. and *Chroococcidiopsis thermalis*, to act as biosorbents for pharmaceutical compounds such as paracetamol, diclofenac, and ibuprofen. The ability of cyanobacteria to metabolise these pharmaceuticals presents a promising approach for their removal from wastewater. However, this research also highlights important challenges, such as the potential for pharmaceuticals to act as unintended nutrient sources, leading to enhanced cyanobacterial growth and the risk of harmful algal blooms if not effectively removed by wastewater treatment systems.

Due to the variable physiology of these organisms, their impact is not uniform. The species *Anabaena* sp. exhibited significantly greater sensitivity in growth and metabolism under the influence of the tested compounds, as indicated by accelerated growth and increased production of photosynthetic pigments. This species also showed high efficiency in the biosorption of paracetamol and diclofenac, with minimal adsorption of these compounds on the cell surfaces. *Chroococcidiopsis thermalis* exhibited slightly higher biosorption efficiency but significantly less sensitivity. It was also proven that the tested pharmaceuticals can be effectively removed at even micromolar concentrations (over 1000 times). It was also confirmed that the removal process favours the hydrophilic compound (paracetamol) over the two hydrophobic compounds (diclofenac and ibuprofen). This metabolic flexibility reduces the concentrations of pharmaceutical residues in water bodies and offers a potential bioremediation strategy for cleaning polluted environments. Furthermore, for the first time, it has been proven that *Anabaena* sp. can effectively transform DCF into its dimer—the known metabolite DCF-2,5-IQ.

Although many studies have examined the biosorption capabilities of a variety of microorganisms, this research specifically targeted *Anabaena* sp. and *Chroococcidiopsis thermalis*, two cyanobacterial species with unique environmental traits. *Anabaena* sp. is particularly notable for its role in nitrogen fixation, while *Chroococcidiopsis thermalis* is recognised for its remarkable resilience in extreme conditions. By focusing on these lesser-studied species, this research provides valuable insights into their ability to metabolise and remove pharmaceutical compounds such as paracetamol, diclofenac, and ibuprofen. This not only enhances our understanding of their biosorption efficiency but also highlights the potential of cyanobacteria in bioremediation applications. Future research could build on these findings by exploring a broader range of cyanobacterial species or comparing the performance of different biosorbents to develop more effective bioremediation strategies for wastewater treatment.

While biosorbents, including cyanobacteria, show great potential for use in wastewater treatment (WWT) systems to remove pharmaceutical contaminants, this study also indicates that, in some cases, pharmaceuticals can act as a nutrient source for cyanobacteria, potentially stimulating their growth. This is a positive factor when contained within WWT systems, as cyanobacteria can effectively metabolise and break down pharmaceutical compounds, contributing to reducing these pollutants. However, pharmaceutical residues in the environment after incomplete removal by WWT processes could present significant ecological risks. If released into natural water bodies, these pharmaceuticals may unintentionally provide cyanobacteria with a nutrient source that promotes their growth, similar to how phosphonates can fuel algal blooms. This can lead to oxygen depletion and other disruptions in aquatic ecosystems, ultimately threatening biodiversity and water quality. This phenomenon underscores the importance of carefully monitoring pharmaceutical residues in aquatic systems to prevent ecological disruptions.

One limitation of this study is the focus on short-term exposure and laboratory conditions, which may not fully capture the complexity of environmental systems. Future research should investigate the long-term ecological effects of pharmaceutical-enhanced cyanobacterial growth, particularly in natural water bodies where cyanobacteria may proliferate due to pharmaceutical contamination. Additionally, the potential toxicity of pharmaceutical metabolites, such as diclofenac dimers, should be explored further to ensure that biotransformation products do not pose a new set of environmental risks.

While this study focused on the biosorption and biotransformation abilities of cyanobacteria in the presence of pharmaceuticals, it is important to acknowledge that a comprehensive toxicological evaluation was beyond the scope of this work. Future studies should address the toxic effects of pharmaceuticals and their metabolites on both cyanobacteria and other aquatic organisms, in accordance with OECD guidelines. This should include determining EC50 values through a series of concentration exposures to better quantify the toxicity of the tested compounds and their transformation products. Such data would provide valuable insights into the ecological risks associated with pharmaceutical contamination and the biotransformation processes studied here.

To better simulate real-world environmental conditions, future studies should explore testing cyanobacteria in systems that mimic more natural environments. One such approach could involve the use of test tanks or bioreactors, where cyanobacteria can be grown under conditions closer to those found in surface waters. These systems would allow for the controlled study of cyanobacterial growth, biosorption efficiency, and pharmaceutical biotransformation in environments with more representative nutrient levels and environmental stressors. Additionally, scaling up to a semi-preparative level using bioreactors or experimental tanks containing surface water could provide valuable insights into the potential of cyanobacteria for practical applications in bioremediation under real environmental conditions.

## Data Availability

Data will be available upon request.
